# Metagenomic analysis of rhizosphere microbiome provides insights into occurrence of iron deficiency chlorosis in field of Asian pears

**DOI:** 10.1186/s12866-021-02432-7

**Published:** 2022-01-08

**Authors:** Bing Jia, Xiao Chang, Yuanyuan Fu, Wei Heng, Zhenfeng Ye, Pu Liu, Li Liu, Yosef Al Shoffe, Christopher Brian Watkins, Liwu Zhu

**Affiliations:** 1 School of Horticulture, Anhui Agricultural University, Hefei, 230036 Anhui P.R. China; 2 College of Agriculture and Life Sciences, Cornell University, Ithaca, NY 14853 USA

**Keywords:** Pear, Fe-deficiency chlorosis, Rhizosphere soil, Microbiome, Metagenomic analysis

## Abstract

**Background:**

Fe-deficiency chlorosis (FDC) of Asian pear plants is widespread, but little is known about the association between the microbial communities in the rhizosphere soil and leaf chlorosis. The leaf mineral concentration, leaf subcellular structure, soil physiochemical properties, and bacterial species community and distribution had been analysed to gain insights into the FDC in Asian pear plant.

**Results:**

The total Fe in leaves with Fe-deficiency was positively correlated with total K, Mg, S, Cu, Zn, Mo and Cl contents, but no differences of available Fe (AFe) were detected between the rhizosphere soil of chlorotic and normal plants. Degraded ribosomes and degraded thylakloid stacks in chloroplast were observed in chlorotic leaves. The annotated microbiome indicated that there were 5 kingdoms, 52 phyla, 94 classes, 206 orders, 404 families, 1,161 genera, and 3,043 species in the rhizosphere soil of chlorotic plants; it was one phylum less and one order, 11 families, 59 genera, and 313 species more than in that of normal plant. Bacterial community and distribution patterns in the rhizosphere soil of chlorotic plants were distinct from those of normal plants and the relative abundance and microbiome diversity were more stable in the rhizosphere soils of normal than in chlorotic plants. Three (*Nitrospira defluvii*, *Gemmatirosa kalamazoonesis*, and *Sulfuricella denitrificans*) of the top five species (*N. defluvii*, *G. kalamazoonesis*, *S. denitrificans*, *Candidatus Nitrosoarchaeum koreensis*, and *Candidatus Koribacter versatilis*). were the identical and aerobic in both rhizosphere soils, but their relative abundance decreased by 48, 37, and 22%, respectively, and two of them (*G. aurantiaca* and *Ca. S. usitatus*) were substituted by an ammonia-oxidizing soil archaeon, *Ca. N. koreensis* and a nitrite and nitrate reduction related species, *Ca. K. versatilis* in that of chlorotic plants, which indicated the adverse soil aeration in the rhizosphere soil of chlorotic plants. A water-impermeable tables was found to reduce the soil aeration, inhibit root growth, and cause some absorption root death from infection by *Fusarium solani*.

**Conclusions:**

It was waterlogging or/and poor drainage of the soil may inhibit Fe uptake not the amounts of AFe in the rhizosphere soil of chlorotic plants that caused FDC in this study.

**Supplementary Information:**

The online version contains supplementary material available at 10.1186/s12866-021-02432-7.

## Background

China is the largest Asian pear fruit producing country in world with annual production of 16,200 k tones, accounting for 68.2% of the world production [1]. Over the last three decades, poor agricultural practices, such as unsustainable fertilization and herbicide application, have resulted in the increasing occurrence of Fe-deficiency chlorosis (FDC) and associated economic losses. Fe is essential both for productivity and quality of plants, because it is associated with photosynthetic efficiency [2]. Fe-deficiency is one of the most common nutrient constraints that is associated with development of chlorosis of plant leaves growing in calcareous and/or alkaline soils [3–6], although manganese, zinc, or calcium deficiencies in the plant will also cause chlorosis [7, 8]. Fe is also necessary for many enzyme functions involved with plant metabolism [9, 10]. Dicots respond to Fe-deficiency by releasing protons into the soil environment and increasing the reducing capacity of the rhizodermal cells [11]. Fe can only be absorbed as an available ferrous iron (Fe^2+^), a soil pH in the range of 7.5-8.5 resulting in low solubility and slow dissolution kinetics of Fe-bearing minerals [12, 13].

Microbial community assembly in the rhizosphere is governed by abiotic and biotic factors, such as soil type, climate change, anthropogenic activities, plant host genotype/cultivar, and pathogenic and mutualistic–symbiotic organisms [14]. In natural ecosystems, the plant species is likely to be a more important determinant of the rhizosphere microbial community than soil type [15]. In contrast, the importance of the rhizosphere microbiota for plant growth and health in agricultural ecosystems is lower compared with that in natural ecosystems owing to the input of fertilizers and pesticides [16]. Microbes have developed various adaptations to thrive in the rhizospheric soil and the host genotype accounts for approximately 5.7% of the variance in the rhizosphere microbiome composition [17, 18].

Progress has been made in the role of soil microorganisms in plant mineral nutrition [19] and the knowledge on composition of rhizospheric microbiota and their dynamics [20]. The plant microbiome contains pathogens, beneficial and neutral microbes, all of which contribute to host fitness and adaptability to the environment [21]. Plant hosts and their developmental stages play an important role in shaping the structure of rhizosphere microbiome [22, 23], most probably by root exudates [24]. Drought also shifts the composition of root microbial communities in many grass species [25, 26]. Host genetic influences on the richness of the microbiomes in rhizosphere soil were shown for maize [27–29]. The rhizosphere microbiome of cotton varied with soil type, genotype and developmental stage [30]. However, whether the bacterial composition in rhizosphere soil is altered to cope with the incidence of FDC in Asian pears is unknown.

The objective of this work was to investigate the differences of subcellular structure and Fe concentration level in Asian pear leaves with and without Fe-deficiency chlorosis.

## Results

### Mineral element content and subcellular structure of leaves

The total ferric iron (TFe) concentrations in ‘Whangkeumbea’ pear leaves with severe, moderate, and mild Fe-deficiency was 91.3, 125.4, and 134.4 mg/kg, respectively, compared with that (328.8 mg/kg) in leaves from normal plant. The critical value of TFe in ‘Whangkeumbea’ pear leaves to Fe-deficiency chlorosis was 150 mg/kg, although there was no significant deference of TFe concentrations between the leaves with moderate and mild Fe-deficiency (Table S1).

Correlation analysis of mineral contents showed that the content of TFe in leaves with Fe-deficiency was positively correlated with the contents of total K, Mg, S, Cu, Zn, Mo and Cl, but negatively correlated with Ca content (Table S2). Therefore, the different degrees of Fe-deficiency chlorosis varied greatly with not only the Fe content itself but also with most of other elements in the leaves.

**Fig. 1:**
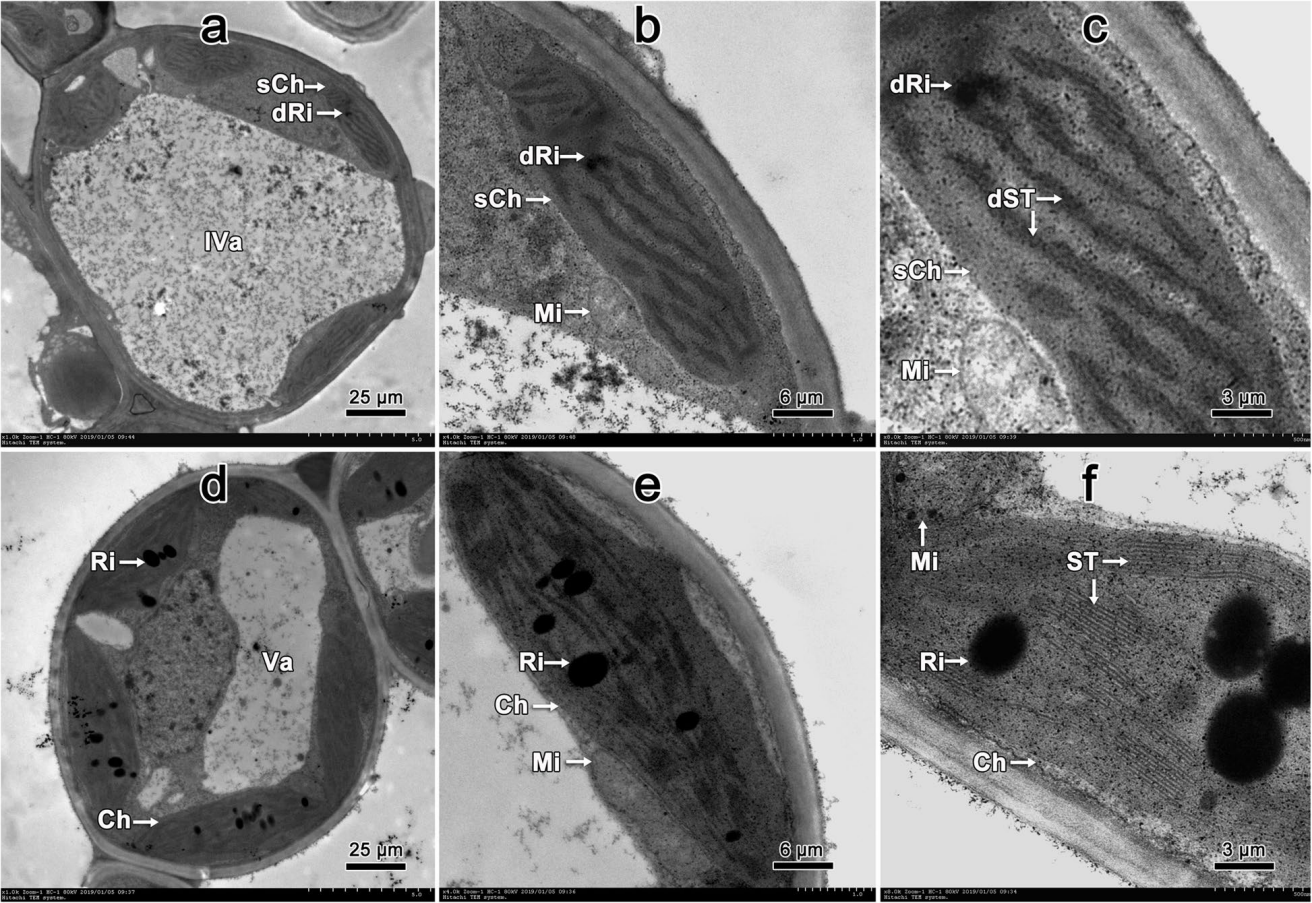
Subcellular structure of leaf from chlorotic and normal plants observed by transmission electron microscope. Degraded ribosome (dRi), destroyed stack of thylakloid (dST) in smaller chloroplast (sCh), larger vacuole (lVa), and normal mitochondria (Mi) found in the cell of leaf with the moderate Fe-deficiency from chlorotic plants (**a**, **b**, and **c**). Normal ribosome (Ri), stack of thylakloid (ST), chloroplast (Ch), vacuole (Va) and Mi observed in the cell of leaf from normal plants (**d**, **e**, and **f**). The magnifications (×1.0k, ×4.0k, and ×8.0k) and scale bars (25 μm, 6 μm and 3 μm) were at the lower right side of each panel, respectively

Transmission electron microscope (TEM) observation revealed that the moderate chlorotic leaves had smaller chloroplasts and larger vacuoles compared with the subcellular structure of normal leaves. Also, degraded ribosomes, degraded stacks of thylakloids in the smaller chloroplast, and normal mitochondria were observed on the transverse section of chlorotic leaves (Fig. 1a, b and c). In contrast, normal ribosomes, stacks of thylakloids in chloroplast, and mitochondria, were found in normal leaves (Fig. 1d, e and f).

### Physicochemical properties of the rhizosphere soil

**Fig. 2:**
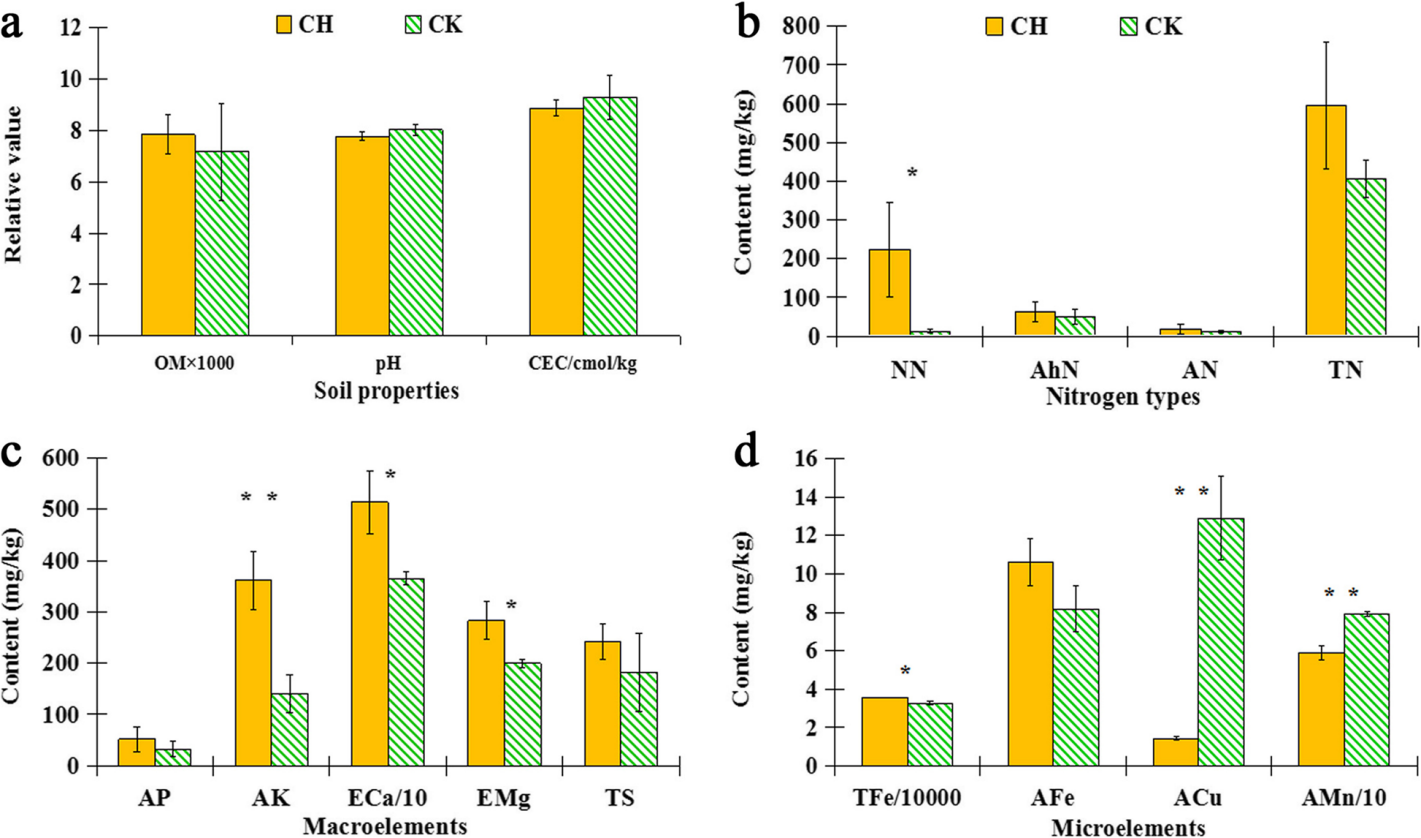
Physiochemical properties in the rhizosphere soil of Fe-deficiency chlorosis (CH) and normal plants (CK). **a** Organic matter (OM), pH value, cation exchange capacity (CEC). **b** Nitrate nitrogen (NN), alkali hydrolysable nitrogen (AhN), amonium nitrogen (AN), and total nitrogen (TN) contents. **c** Available phosphorus (AP), available potassium (AK), exchangeable calcium (ECa), exchangeable magnesium (EMg), and total sulfur (TS) content. **d** Total ferrum (TFe), available ferrous (AFe), available copper (ACu), and available manganese (AMn) content. Error bars indicate ± standard error (SE, *n* = 3), * *P*-value ≤ 0.05, and ** *P*-value ≤ 0.01

No significant differences of Organic matter (OM), pH values, and cation exchange capacity (CEC) in the rhizosphere soil between chlorotic and normal plants were detected (Fig. 2a). However, nitrate nitrogen (NN), available potassium (AK), exchangeable calcium (ECa), and exchangeable magnesium (EMg) in the rhizosphere soil of chlorotic plants were higher than that of normal plants, but there was no significant differences of the total nitrogen (TN), alkaline-hydrolysable nitrogen (AhN), ammonium nitrogen (AN), available phosphorus (AP), and total sulfur (TS) contents in the rhizosphere soil between the two plant types (Fig. 2b and c).

ACu and AMn in the rhizosphere soil of chlorotic plants were lower than that of normal plants. It was interesting that the contents of TFe and AFe in the rhizosphere soil of chlorotic plants were higher than in that of normal plants, although there was no significant difference of the AFe content in the rhizosphere soil between the two plant types (Fig. 2d).

### Number of genes and gene ontology (GO) function and pathway enrichment in the rhizosphere soil

After the metagenomic sequencing of the six samples of rhizosphere soil (CH1, GH2, and CH3 from Fe-deficiency chlorosis plants and CK1, CK2, and CK3 from normal control plants), an average of 7.12 Gbp clean data was generated per sample, and a total of 166, 658 non-redundant genes were identified. The average of total and annotated genes in the rhizosphere soil of chlorotic plant was 84,605 and 64,317, respectively, which was 18,779 and 13,182 genes more than that of normal plants, respectively (Supplementary Table S3).

**Fig. 3:**
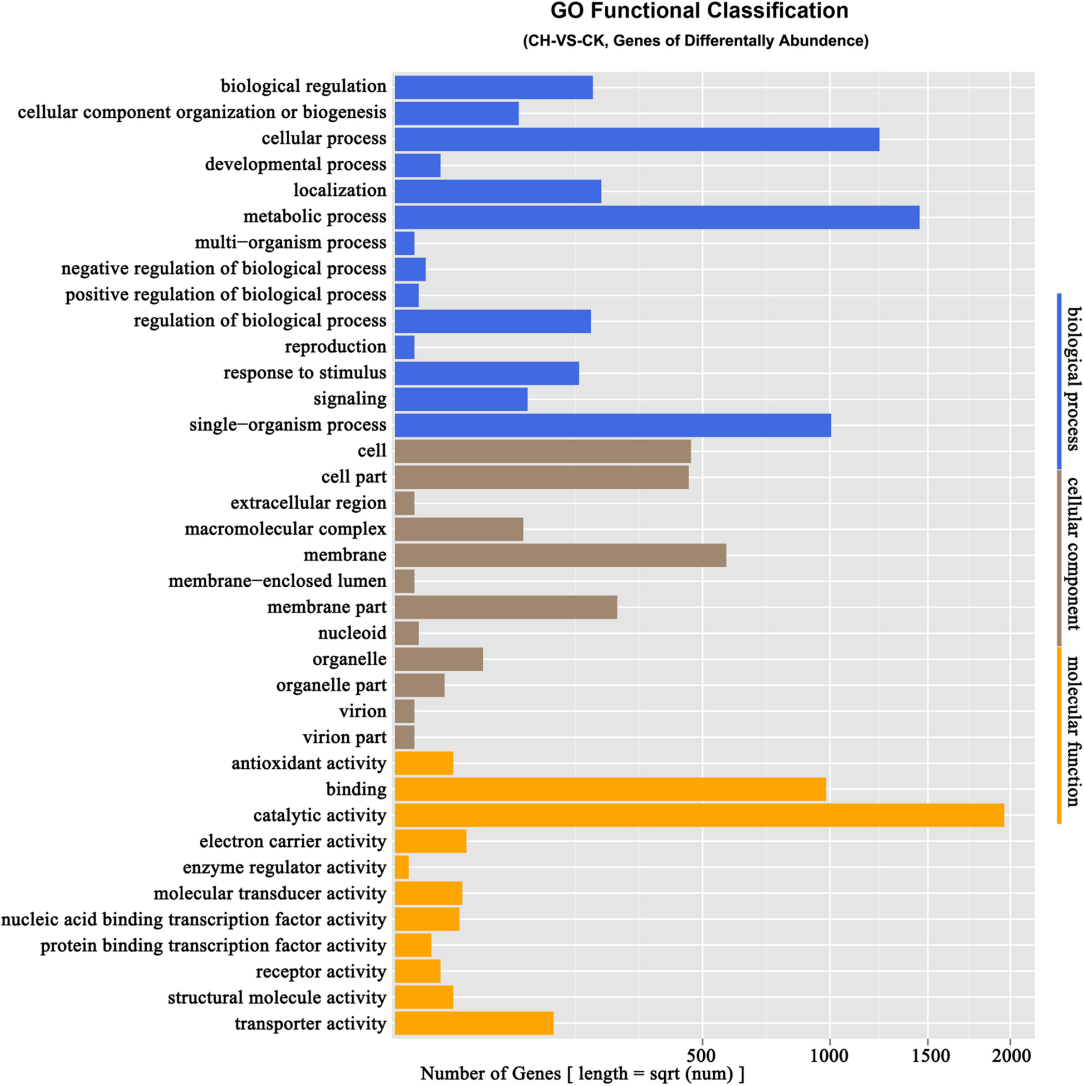
GO functional classification of differently expressed genes between the rhizosphere soil of chlorosis and normal plants. CH. rhizosphere soil of chlorotic plants; CK. rhizosphere soil of normal plant

Quantitative and differential analysis of gene abundance in CK compared with CH identified 5,552 total differentially expressed genes (DEGs); of these 4,624 genes were high-abundance and 928 genes were low-abundance in the rhizosphere soil of chlorotic plants. The DEGs between the rhizosphere soil of chlorotic and normal plants were categorized into 37 GOs. Among the biological process categories, those associated with metabolic processes was the largest class, including 1,422 genes and accounting for 25.6% of the total DEGs), followed by cellular processes (1,214; 21.9%), single-organism processs (999; 18.0%), localization (224; 4.0%), biological regulation (207; 3.7%), and regulation of biological processes (203; 3.7%). In the molecular function category, 1,961 (35.3%) and 982 (17.7%) DEGs were classified into catalytic activity and binding, respectively, followed by transporter activity (133; 2.4%). With respect to the cellular component category, membrane were the largest class (580; 10.4%), followed by cell (463; 8.4%), cell part (456; 8.2%), membrane parts (261; 4.7%), and macromolecular complex (87; 1.6%) (Fig. 3).

**Fig. 4:**
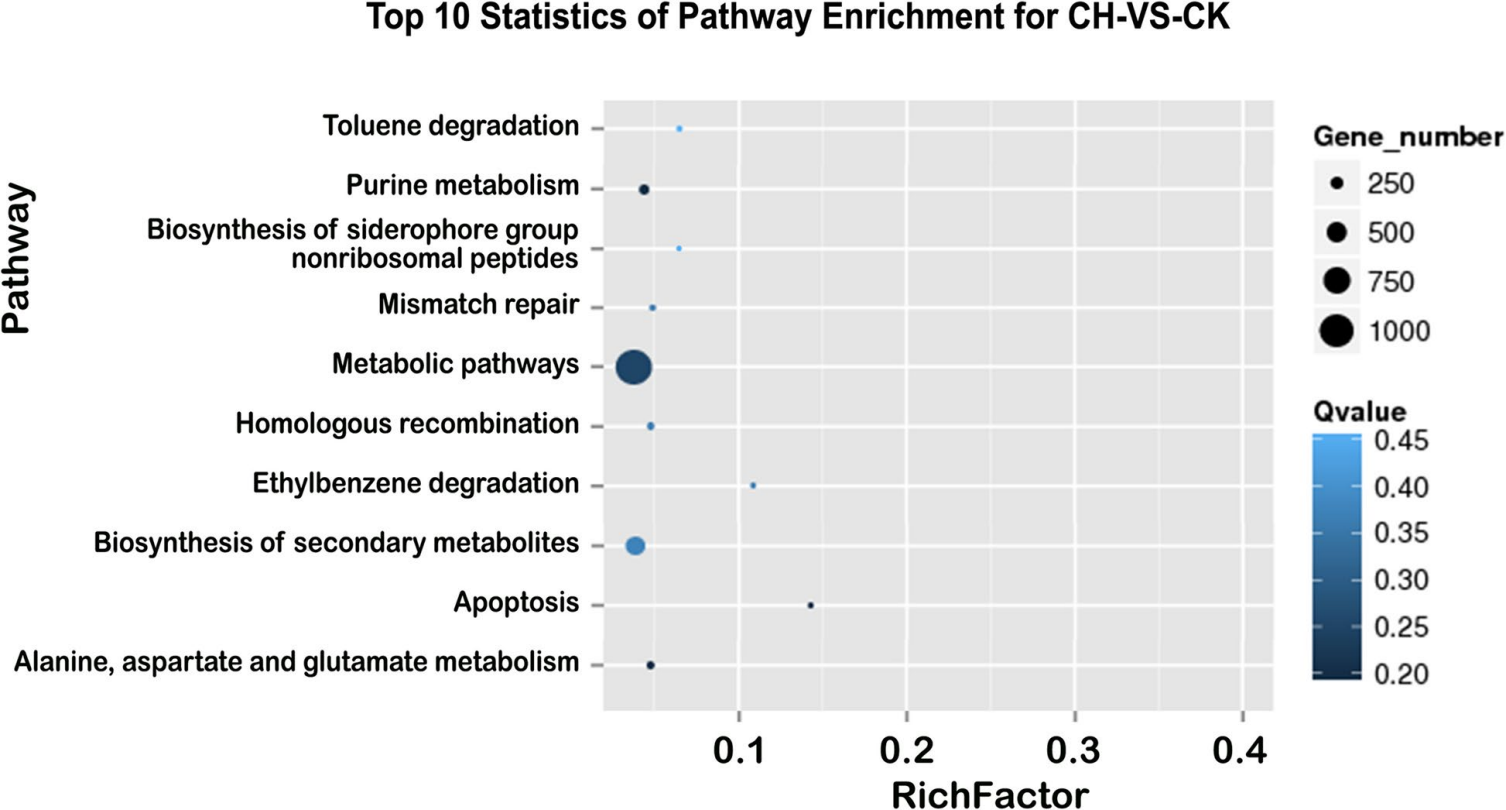
Top 10 pathways enriched for the differently expressed genes between rhizosphere soil of chlorosis and normal plants. CH. rhizosphere soil of chlorotic plants; CK. rhizosphere soil of normal plants

After the DEGs were subjected to Kyoto Encyclopedia of Genes and Genomes (KEGG) enrichment analysis, there were 10 pathways significantly enriched in CH vs CK (*P* value<0.05). Compared with that of normal plants, the DEGs enriched in the rhizosphere soil of chlorotic plants were involved mainly in metabolic pathways (1,090 DEGs; 33.94%), biosynthesis of secondary metabolites (495 DEGs; 15.41%), purine metabolism (159 DEGs; 4.95%), alanine, aspartate and glutamate metabolism (77 DEGs; 2.40%), homologous recombination (47 DEGs; 1.46%), and mismatch repair pathways (42 DEGs; 1.31%) (Supplementary Table S4; Fig. 4).

### Microbiota in the rhizosphere soil

The community compositions of annotated microbiome indicated that there were 5 kingdoms, 52 phyla, 94 classes, 206 orders, 404 families, 1,161 genera, and 3,043 species in the rhizosphere soil of chlorotic plants; it was one phylum less and one order, 11 families, 59 genera, and 313 species more than in that of normal plant (Supplementary Table S3).

**Fig. 5:**
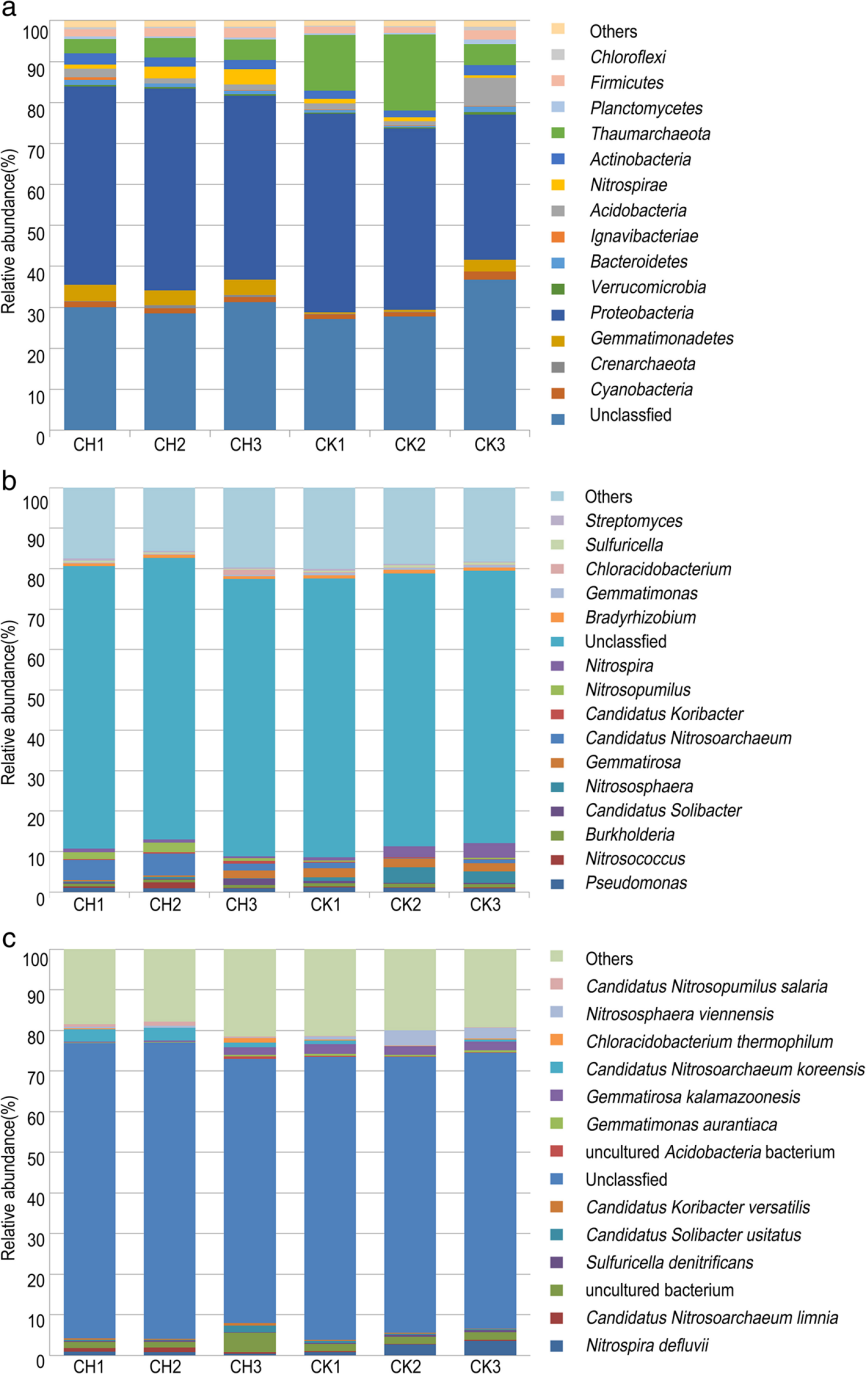
Relative abundance of microbiota in the rhizosphere soil of chlorotic and normal plants. CH1-3. rhizosphere soil from chlorotic plants; CK1-3. rhizosphere soil from normal plants. Presented phylum (**a**), genus (**b**), and species (**c**) were defined as having a relative abundance greater than 0.5% in any one sample from chlorotic and normal plants. Relative abundance less than 0.5% in all samples at each taxonomic level were combined into the others

Fifteen phyla were observed at relatively high abundance, which was defined as having a relative abundance greater than 0.5% in any one sample from chlorotic and normal plants, beside the unclassified (accounting for 30.6 and 29.9% of the total in chlorotic and normal plants, respectively) and the others (1.4%; 1.5%). Notably, the top five phyla dominated in the rhizophere soil of chlorotic plants accounted for 62.1% of the total: Proteobacteria (42.7%), Thaumarchaeota (12.4%), Acidobacteria (3.2%), Actinobacteria (2.1%), and Firmicutes (1.8%); while that of normal plant accounted for 60.6% of the total: Proteobacteria (47.5%), Thaumarchaeota (4.4%), Gemmatimonadetes (3.8%), Nitrospirae (2.5%) and Actinobacteria (2.4%). Among these top five phyla, three of them were identical and two of them were different (Fig. 5a; Table S5). At the level of genus, *Nitrososphaera* (2.5%), *Nitrospira* (2.4%), *Gemmatirosa* (2.2%), *Pseudomonas* (1.0%), and *Burkholderia* (0.8%) were the top five genera from sixteen ones with an average relative abundance greater than 0.5% in any one sample of the rhizosphere soil from normal plant; while that from chlorotic plants *Candidatus Nitrosoarchaeum* (4.0%), *Nitrosopumilus* (1.6%), *Pseudomonas* (0.9%), *Gemmatirosa* (0.8%), and *Nitrospira* (0.7%) were the top five genera, two of them were different and three of them were identical but at different relative abundance (Fig. 5b).

Compared with the rhizosphere soil of normal plant, the relative abundance of the top 12 species in the rhizosphere soil of chlorotic plants showed that *Candidatus Nitrosoarchaeum koreensis* was the most dominant species with the average relative abundance of 2.40% and an increase of 80.0%, followed by *Gemmatirosa kalamazoonesis* (0.85%; and 60.8%), *Candidatus Nitrosoarchaeum limnia* (0.84%; and 78.0%), *Nitrospira defluvii* (0.74%; and 69.0%), *Candidatus Solibacter usitatus* (0.67%; and 44.5%), *Candidatus Nitrosopumilus salaria* (0.58%; and 84.5%), *Chloracidobacterium thermophilum* (0.51%; and 62.2%), *Candidatus Koribacter versatilis* (0.42%; and 59.7%), *Nitrososphaera viennensis* (0.32%; and -87.1%), *Sulfuricella denitrificans* (0.29%; and 34.7%), uncultured *Acidobacteria bacterium* (0.26%; and 53.9%), and *Gemmatimonas aurantiaca* (0.20%; and 61.43%), beside the uncultured bacterium (2.47%; and 28.0%), the others (19.30%; and 4.2%), and the unclassified species (70.16%; and 2.3%). For in CK vs CH the relative abundance was different only for *C. N. koreensis*, *G. kalamazoonesis*, *C. N. limnia*, *G. aurantiaca*, and *C. N. salaria* (Fig. 5c; Table S6).

### Characterization of bacterial communities and simplified species compositions

**Fig. 6:**
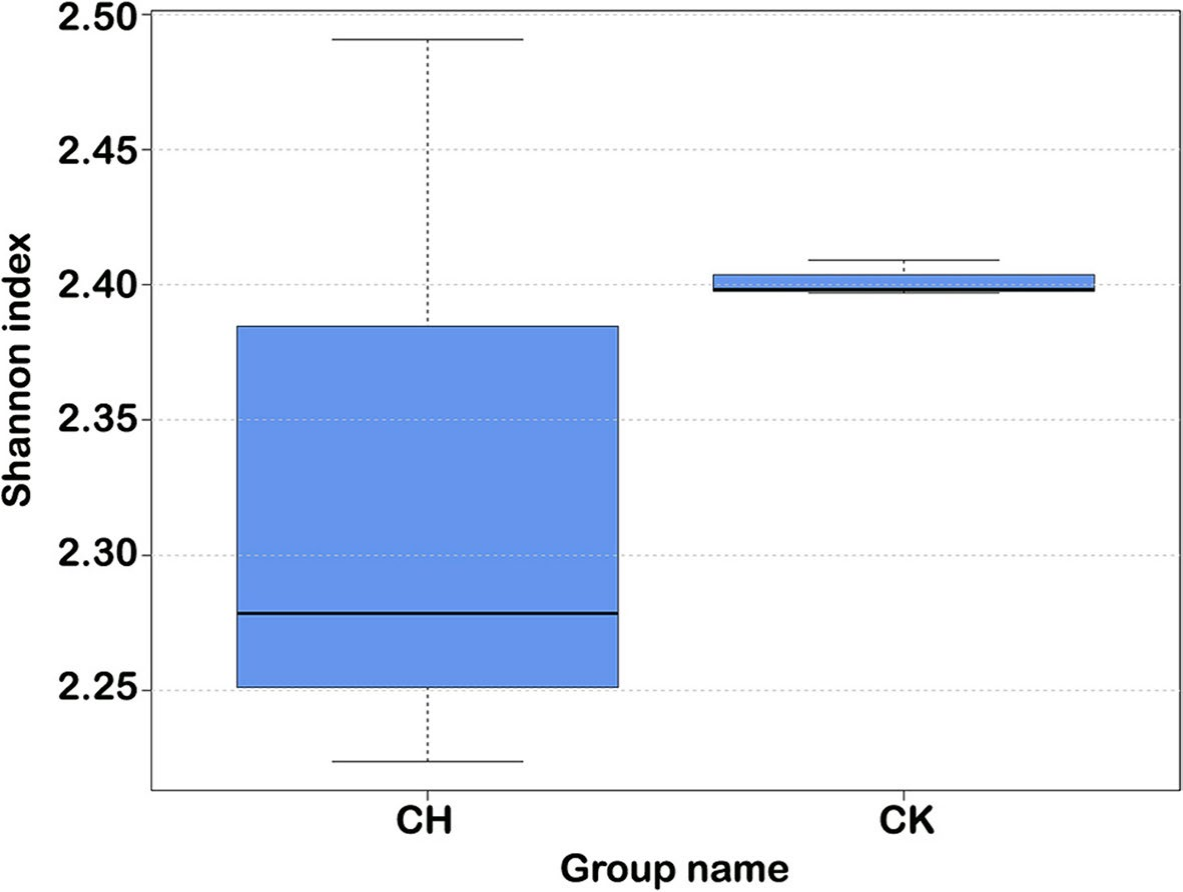
Shannon index of microbiome in the rhizosphere soil of chlorotic and normal plants. CH. Rhizosphere soil of chlorotic plants, CK. Rhizosphere soil of normal plants. The larger the Shannon index, the better the sample uniformity. In the case of the same species richness, the greater the uniformity of each species in the community, the greater the diversity of the community

Species diversity analysis showed that the bacterial community in the rhizosphere soil of normal plant had a higher and less variable Shannon index (Mean ± SE = 2.403 ± 0.006) than in that of chlorotic plant (Mean ± SE = 2.330 ± 0.142). It demonstrated that the rhizosphere soil of normal plants had greater diversity of the bacterial community than that of chlorotic plants (Fig. 6).

**Fig. 7:**
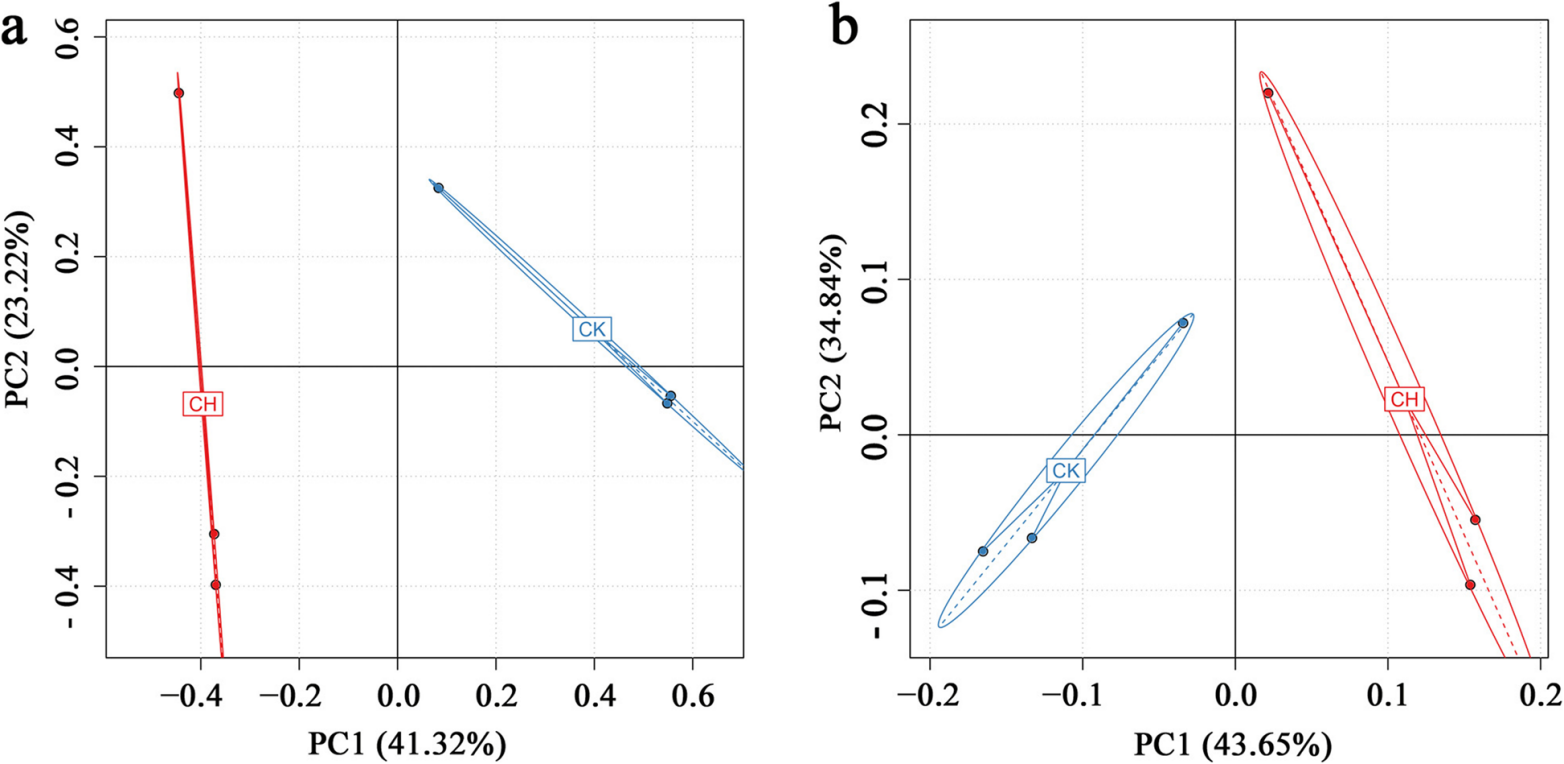
Principle component analysis based on the expressed genes annotated and abundance of bacterial species in the rhizosphere soil of chlorotic and normal plants. Each round-point represents a sample replicate, the abscissa is the value of principal component 1, and the ordinate is the value of principal component 2. **a** Expressed genes annotated; **b** Abundance of bacterial species; CH. Rhizosphere soil of chlorotic plants, CK. Rhizosphere soil of normal plants

Principle component analysis (PCA) revealed that a relatively large portion of the total variance contributed to the first two PCs based on expressed genes annotated (PC-1 = 41.32% and PC-2 = 23.22%) and abundance of taxonomic species (PC-1 = 43.65% and PC-2 = 34.48%), respectively. The two distinctive groups (CK1-3, normal plants; CH1-3, chlorotic plants) was formed, not only by the number of expressed genes annotated (Fig. 7a), but also by the abundance of taxonomic species (Fig. 7b).

**Fig. 8:**
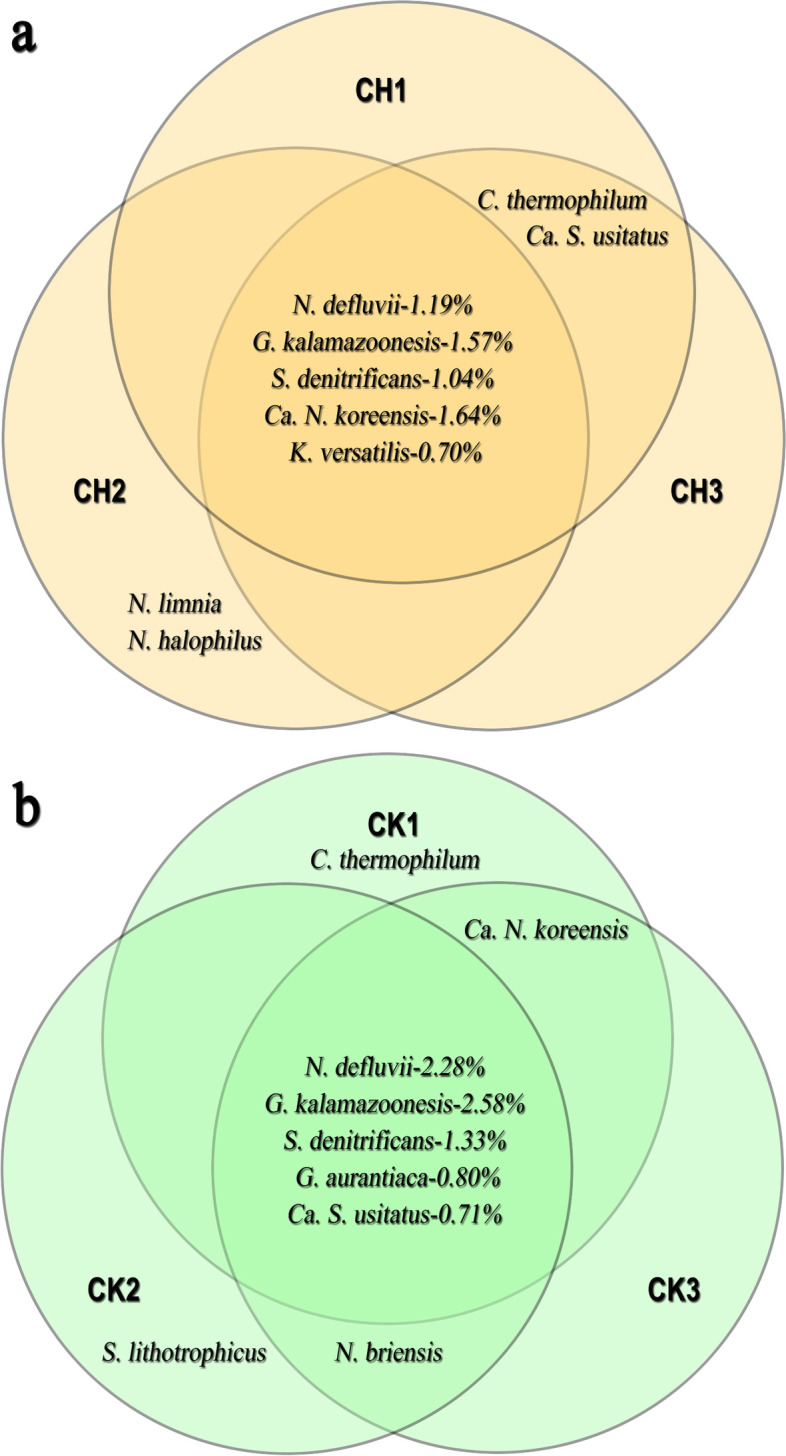
Simplified species composition in rhizosphere soil of chlorotic (**a**) and normal (**b**) plants. CH1-3. Rhizosphere soil of chlorotic plants, CK1-3. Rhizosphere soil of normal plants. The five dominant species for CH1-3 were *Nitrospira defluvii*, *Gemmatirosa kalamazoonesis*, *Sulfuricella denitrificans*, *Candidatus Nitrosoarchaeum koreensis*, and *Candidatus Koribacter versatilis*. The five dominant species for CK1-3 were *Nitrospira defluvii*, *Gemmatirosa kalamazoonesis*, *Sulfuricella denitrificans*, *Gemmatimonas aurantiaca*, and *Candidatus Solibacter usitatus*

By synthetic analysis of the species presented in all samples with relative abundance higher than 0.6%, we obtained the most simplified communities. In the rhizosphere soil of chlorotic plants the top five species were *Nitrospira defluvii*, *Gemmatirosa kalamazoonesis*, *Sulfuricella denitrificans*, *Candidatus Nitrosoarchaeum koreensis*, and *Candidatus Koribacter versatilis*), while in that of normal plants the top five species were *N. defluvii*, *G. kalamazoonesis*, *S. denitrificans*, *Gemmatimonas aurantiaca*, and *Candidatus Solibacter usitatus*. Three of them, i.e. *N. defluvii*, *G. kalamazoonesis*, and *S. denitrificans* were the same in both rhizosphere soils, but their relative abundance decreased by 48, 37, and 22%, respectively, and two of them (*G. aurantiaca* and *Ca. S. usitatus*) were substituted by an ammonia-oxidizing soil archaeon, *Ca. N. koreensis* and a nitrite and nitrate reduction related species, *Ca. K. versatilis* in that of chlorotic plants (Fig. 8).

### Water-impermeable table formed by silt stratification inhibit root growth

A water-impermeable table formed by silt stratification was found in the chlorotic plants but not the normal plants. As for root growth, it is normal in the upper layer of soil and some absorption roots in the middle and lower layers of soil were decayed (Supplementary Fig. S2).

A total of five fungi species were isolated from the roots of chlorotic plants; four of them were pathogenic fungi and one of them was non-pathogenic (*Nigrospora oryzae*). In contrast, no pathogenic fungi were isolated and only one non-pathogenic fungus (*Neurospora intermedia*) was identified from the roots of normal plant. Four bacterium species were identified in normal plants, and three bacterium species were found in the roots of chlorotic plants, but none of them was pathogenic ones (Table 1).

**Table 1:** Fungal and bacterial species identified in the roots of chlorotic and normal plants

Microorganism	Roots of Chlorotic Plant	Roots of Normal Plant
Non-pathogenic Fungus	*Nigrospora oryzae*	*Neurospora intermedia*
Pathogenic Fungus	*Fusarium solani*	*--*
	*Fusarium phaseoli*	*--*
	*Pythium spinosum*	*--*
	*Ilyonectria macrodidyma*	*--*
Non-pathogenic Bacterium	*Paenibacillus lautus*	*Bacillus flexus*
	*Bacillus niacini*	*Bacillus pumilus*
	*Bacillus gibsonii*	*Paenibacillus sp.*
	*--*	*Lysinibacillus fusiformis*

## Discussion

### Leaf structural changes associated with Fe-deficiency chlorosis

Fe-deficiency enlarged the epidermal cells and reduced the guard cell size, bundle sheaths appeared disorganized, and the mesophyll was more compacted and less porous compared to normal (healthy) pear leaves [31]. However, there have not been previous reports of ribosomal degradation before the stacks of thylakloids began to destruct in chloroplast observed on the ultrathin transverse section of leaves with FDC.

Inorganic cation and anion concentrations in apoplastic fluid of sugar beet leaves increased with Fe deficiency, and the largest increase in apoplastic fluid concentrations with Fe deficiency was 20-fold for Ca, followed by 3.5-fold for Mg, and 1.7-fold for K [32]. Our results show that ‘Whangkeumbea’ pear leaves with Fe-deficiency were negatively correlated only with Ca content, but not those of Mg and K. In *Arabidopsis* seeds, only 5% of Fe is ferritin-bound and its main storage compartment is the vacuole [33]; up to 80% of the cellular Fe was found in the chloroplast, which is consistent with its major function in photosynthesis [34]. But the mechanism by which chloroplasts become smaller while the vacuole increases in size of leaves with FDC remains to be clarified.

### Key factor related to the Fe-deficiency chlorosis

In pear plants, Fe-deficiency chlorosis was associated with decreased leaf chlorophyll concentrations, fruit number, and total fruit weight per tree by 36, 72, and 64%, respectively [35, 36]. Fe-deficiency may occur because there is an insufficient Fe amount in the soil [8, 34]. Fe is unavailable because of high pH in alkaline soils [7], or the Fe may not be absorbed due to the root damage by soilborne diseases [37]. However, in our study, TFe and AFe contents in the rhizosphere soil of chlorotic plants were higher than those of normal plants, and there were no significant differences of pH values in the rhizosphere soil between chlorotic and normal plants. It was demonstrated that Fe concentration, and high pH value in the soil were not the possible causes of Fe-deficiency chlorosis in this study.

Although Fe-deficiency chlorosis has a typical symptom, the loss of green color only on leaf interveinal tissue, the level of total Fe in Asian pear leaves attributed to Fe-deficiency chlorosis varied with cultivars, the critical value of ‘Cuiguan’ pear leaves is 70 mg/kg [38], and which of ‘Yali’ pear and ‘Niitaka’ pear leaves is around 113 mg/kg and 97 mg/kg, respectively [39]. The critical value of total Fe in ‘Whangkeumbea’ pear leaves is 150 mg/kg in the present study. Otherwise, there is no significant difference between the total Fe contents of the moderate and mild Fe-deficiency leaves (Table S1), but the phenotypes have showed different symptoms, demonstrating the Fe contents is not strictly correlated to the extent of chlorosis. The content of TFe in leaves with Fe-deficiency was positively correlated with the contents of total K, Mg, S, Cu, Zn, Mo and Cl, but negatively correlated with Ca content, when Fe-deficiency chlorotic plants occurred in field of Asian pears (Table S2).

Plants release one proton (H^+^) for every ammonium (NH_4_^+^) taken up resulting in a reduction pH in rhizosphere. When supplied with nitrate (NO_3_^-^), the plant releases bicarbonate, which will increase the rhizosphere pH. These changes in pH can influence Fe availability in the soil [11]. The NO_3_^-^ content in the rhizosphere soil of chlorotic plants was higher but the pH value was lower than that of normal plant. It is possible that the amount of NO_3_^-^ supply was insufficient to increase the pH in rhizosphere soil of chlorotic plants.

The pear plants in the experimental orchard showed the symptom of Fe deficiency chlorosis, and it was proved by leaf analysis and leaf subcellular structure observation. After the soil analysis was done, it was found that there was no deference of AFe concentration between the rhizosphere soils of chlorotic and normal plants, and TFe concentration was even significantly higher in the rhizosphere soils of chlorotic plants than that of normal plants, demonstrating it was Fe uptake of plant root not Fe supply of soil that lead to the Fe-deficiency chlorosis of pear plants in this study. Therefore, the bacterial species community in the rhizosphere soils had been analyzed by metagenomic analysis to ascertain the practical factor inhibiting Fe uptake of these chlorosis plants.

### Bacteria species composition provide insight into potential indication of FDC occurrence

Among the top five bacterial species, three of them (*N. defluvii*, *G. kalamazoonesis*, and *S. denitrificans*) were identical in the rhizosphere soils of chlorotic and normal plants, but with lower relative abundance, two of them (*G. aurantiaca* and *Ca. S. usitatus*) in rhizosphere soils of normal plants were substituted by *Ca. N. koreensis* and *Ca. K. versatilis* in rhizosphere soils of chlorotic plants.

*N. defluvii* is a nitrite-oxidizing bacterium firstly enriched from activated sludge from a municipal wastewater treatment plant [40], which catalyzes aerobic nitrite oxidation and is adapted to live under significant substrate limitation with half-saturation constants (*Km*) 9 μM NO_2_^-^ [41]. The highest proportions of *Gemmatimonadetes* strains were isolated in arid soils, suggesting an adaption to low soil moisture [42]; it is found in a wide variety of environments and is particularly abundant in soils [43]. *G. kalamazoonesis* is a microaerophillic or aerobic bacterium belonging to the phylum *Gemmatimonadetes*. It does not produce catalase, and was isolated initially from agricultural soil in Kalamazoo, Michigan, United States [44]. *S. denitrificans* is a sulfur-oxidizing bacterium isolated from anoxic water of a freshwater lake in Japan [45], which is microaerobic and could utilize NO_3_^-^ as an electron acceptor, reducing it to nitrogen [46].

*G. aurantiaca* was present in all samples of rhizosphere soil of normal plant but was absent in the soil of chlorotic plants. It is an aerobic bacterium isolated first from an anaerobic-aerobic sequential batch reactor operated under enhanced biological phosphorus removal conditions for wastewater treatment [47].

Aerobic bacteria require oxygen to live and grow, while microaerophillic and microaerobic bacteria require very little oxygen to maintain growth. The greatly reduced abundance of the top three bacteria species *N. defluvii*, *G. kalamazoonesis*, and *S. denitrificans* from the rhizosphere soils of chlorotic plants may result from adverse soil aeration. Waterlogging or/and poor drainage of the soil may inhibit Fe uptake. After the field investigations were completed, we found water-impermeable tables formed by silt stratification in middle layer of soil at the chlorotic plants. This would reduce the aeration of middle and lower layers of soil, and result in poor root growth caused mainly by the dominant pathogenic species *F. solani* [37], leading to the Fe-deficiency chlorosis of pear plants in this study.

In general, the primary causes of the formation of water-impermeable silt stratification in sandy soil are flood irrigation, frequent farming of the topsoil and long-term stratification of silt by a rich precipitation without deep ploughing to break the silt layer in several years. However, the third one should be the practical cause of the formation of silt stratification in the present study, because the drip irrigation and cover cropping between rows were applied in this experimental orchard.

### Changes of microbial community and iron availability in rhizosphere soil

*Ca. S. usitatus* is a NO_2_ and NO_3_ reduction species isolated from soil in ryegrass grass/clover pasture in Australia [48], and its genome contains not only *nirA* encoding nitrite reductase but also genes associated this uptake of Fe, including the genes *feoAB*, which encode a high-affinity ferrous (Fe^2+^) iron transporter [49]. *Ca. K. versatilis* is also a NO_2_ and NO_3_ reduction species [50], which contain genes encoding iron permease FTR1 and FTR1 family proteins as well as sequences similar to *fet3p* [49], which encodes a multicopper oxidase involved in FTR1-mediated iron uptake in yeast [51]. *Ca. K. versatilis* and *Ca. S. usitatus*, are present in the rhizosphere soil of chlorotic and normal plants, respectively; their genomes contain genes encoding candidate siderophore receptors and other Fe transporter genes suggesting that they may be involved in Fe^2+^ uptake.

*Ca. N. koreensis* is an ammonia-oxidizing soil archaea belonging to the phylum *Thaumarchaeota*, which is an anaerobic bacterium and ubiquitous microbes in marine, freshwater, and terrestrial environments [52]. However, anaerobic bacteria generate energy by the oxidation of Fe^2+^ compounds to Fe^3+^ [53]. As an anaerobic bacterium, *Ca. N. koreensis* is present mainly in the rhizosphere soil of chlorotic plants but it is absent from that of normal plants. This suggests that it may be involved in decrease of Fe availability in rhizosphere soil for plant absorption, although the concentrations of TFe and AFe tested in this study were higher in the rhizosphere soil of chlorotic plants.

Some bacteria, such as *Ca. K. versatilis* and *Ca. S. usitatus*, can scavenge Fe via excreted siderophores. It still needs to be proven whether or not these bacteria have potential advantage to compete Fe with roots in rhizosphere soil, where Fe is bound to soil colloids and the available concentration is limited to pear plant growth.

## Conclusions

FDC affected Fe content in the leaves as well as most other elements. The higher amounts of TFe and AFe in the rhizosphere soil of chlorotic plants demonstrated it was the Fe uptake and not the influence of pH or the availability of Fe in the soil that caused FDC symptom. The chloroplasts were smaller and the vacuoles larger, and degraded ribosomes and stacks of thylakloids in chloroplast of chlorotic leaves from FDC plant compared with normal plants.

The results of microbiome analyses showed that the rhizosphere soil of normal plants had greater diversity of the bacterial community than that of chlorotic plants, and two distinctive groups (normal plants and chlorotic plants) was formed by PCA using the number of expressed genes and the species abundance.

Among the top five species in the rhizosphere soils of chlorotic and normal plants, three of them (i.e. *N. defluvii*, *G. kalamazoonesis*, and *S. denitrificans*) were the identical and aerobic, but their relative abundance greatly decreased, and two of them (*G. aurantiaca* and *Ca. S. usitatus*) were substituted by an anaerobic species (*Ca. N. koreensis*) and a species scavenging Fe via excreted siderophores (*Ca. K. versatilis*) in that of chlorotic plants. The greatly reduced abundance of aerobic bacteria species and emergence of anaerobic bacteria species in the rhizosphere soils of chlorotic plants implied the soil aeration was at adverse situation.

A water-impermeable table formed by silt stratification was found in middle layer of soil of the chlorotic plants but not in that of normal plants. Some absorption roots in the middle and lower layers of soil died from the soilborne disease mainly caused by the dominant pathogenic species *Fusarium solani* due to the anoxic soil condition.

## Methods

### Leaf sample preparation

Asian pear (*Pyrus pyrifolia* Nakai) plants with or without Fe-deficiency chlorosis were surveyed in successive three years (2016-2018) at the Old Yellow River Valley Orchard (116.92_E; 34.34_N), located in Xiaoxian county, Anhui province, China, where the annual precipitation is about 800 mm. Forty year-old ‘Whangkeumbea’ pear trees grafted onto rootstock of *P. betulaefolia* Bge., grown in sandy loam soils with drip irrigation and cover cropping between rows were used for these experiments. According to the degree of leaf chlorosis, 60 mature leaves with typical iron-deficient symptoms (divided into mild: Fig. S1A, moderate: Fig. S1B, and severe: Fig. S1C) were taken from different plants with chlorosis (Fig. S1D), and control leaves (Fig. S1E) were taken from different normal plants on June 20, 2018 (Fig. S1F), and then, brought back to the laboratory and divided into two groups, one for the leaf transversal sections, and one for cleaning, drying and grinding to determine the contents of N, P, K, Ca, Mg, and S in percentage on a dry weight (DW) basis, and Fe, B, Mn, Cu, Zn, Mo and Cl, expressed as mg·kg^-1^ on a dry weight basis at the Institute of Geology and Mineral Resources, Department of Land and Resources of Anhui Province, Hefei, China. We confirmed that all methods complied with relevant institutional, national, and international guidelines and legislation.

### Soil sample collection

Rhizosphere soil was defined as that tightly bounded to the root surface [16]. Each composite soil sample was collected on August 20, 2018 at the rhizosphere sites from the east, south, west, and north sides, from three replicate trees with leaves exhibiting Fe-deficiency chlorosis (CH 1-3) and normal plants as control (CK1-3). All the samples were frozen in liquid nitrogen, packed into sterile polyethylene bags and immediately transported on ice packs to the laboratory. The soils were sieved through 2 mm mesh to remove visible roots, residues and stones. Each replicate was then divided into two parts: one was stored at 4°C for the analyses of soil physiochemical properties while the other part was stored at -80°C within a week for DNA extraction and metagenomic analysis of the rhizosphere microbiome.

### Leaf subcellular structure observation

The leaf pieces were treated with 2.5% glutaraldehyde solution under vacuum until the leaf pieces sank to the bottom of the bottle, and fixed at 4°C for 24 hours. The pieces were rinsed with 0.1 M pH 7.0 phosphate buffers 3 times for 15 min; fixed with 1% osmium tetroxide for 1-2 h, rinsed 3 for 15 min with 0.1 M pH 7.0 phosphate buffer. After dehydration in a graded ethanol series (50, 70, 90, and 100%) for 10 min at each step, the pieces were embeded in Epon 812 epoxy resin. Cross sections were then taken by a microtome (LKB-V, Uppsala, Sweden) at a thickness of 70-90 nm. Finally, the cross sections were stained with uranyl acetate-lead citrate and imaged with a transmission electron microscope (TEM) (Hitachi 7650, Tokyo, Japan) operating at an accelerating voltage of 80 kV. Images were acquired at the magnifications of 1.0, 4.0, and 8.0 k [54].

### Analyses of soil physiochemical properties

Soil pH was determined with a soil to water ratio of 1:5 using a pH monitor (Orion-868, Thermo-Fisher Scientific, city, USA). The soil was air dried and sieved (1 mm mesh). Organic matter (OM) was determined using the potassium dichromate-sulphuric acid heating method [55], and cation exchange capacity (CEC) was determined by titration with standard sodium hydroxide solution [56]. The total nitrogen (TN) content was analyzed by the Kjeldahl method, while the alkaline-hydrolysable nitrogen (AhN) was measured by the hydrolysis diffusion method [57]. The nitrate nitrogen (NN), ammonium nitrogen (AN), and available phosphorus (AP) were determined using a continuous flow analyzer (AutoAnalyzer 3, Bran+Luebbe, Germany). Available potassium (AK) was determined by flame Photometry (FP8800, Topac Inc., MA, USA) and total sulfur (TS) were determined by combustion (CNS-2000; LECO, St. Joseph, MI, USA). Exchangeable calcium (ECa), exchangeable magnesium (EMg) were determined by atomic absorption spectrophotometry (Hitachi Z-5000 Polarized Zeeman, Tokyo, Japan). The microelements, including total ferric iron (TFe), available Ferrous iron (AFe), available molybdenum (AMo), available boron (AB), available zinc (AZn), available copper (ACu), and available manganese (AMn) were determined by Inductively Coupled Plasma Optical Emission Spectrometer (ICP-OES; Agilent, Santa Clara, CA 95051, USA).

### DNA extraction and sequencing

The genomic DNA was extracted from each sample of rhizosphere soil at The Beijing Genomics Institute (BGI) Co., Ltd (BGI-Shenzhen, Guangdong, China). The sequencing library construction method was optimized based on the Illumina operating instructions. The Covaris focused ultrasound system was used to first break the qualified DNA sample. After the end repair was performed by adding End Repair Mix at 20°C for 30 minutes, the DNA fragment was purified using a PCR purification kit (QIAGEN, Shanghai, China). A base was added to the 3'-end using A-Tailing Mix at 37°C for 30 minutes and then a sequencing adapter was ligated to both ends of the DNA fragment. Fragment selection was performed using 2% agarose gel and QIA quick Gel Extraction kit (QIAGEN), and several cycles of PCR amplification were performed. The PCR product was again purified with 2% agarose gel and the target fragment was recovered using an extraction kit (QIAGEN). Finally, the quality of sample library was controlled and quantified using the Agilent 2100 Bioanaylzer and the ABI StepOne Plus Real-Time PCR System. The qualified library was sequenced using an Illumina HiSeq^TM^ 2000 next-generation sequencer [58, 59] at the BGI facility.

The raw reads produced by the Illumina HiSeq™ 2000 were filtered into clean reads by removing the unqualified reads, which contain more than 10% unknown bases, 15 or more bases aligned to the adaptor sequence, and more than 20% of bases in the total reads having a quality score lower than 15 after sequencing.

### Bioinformatics analysis

The qualified reads were first assembled by using SOAP denovo2 [60] and Rabbit [61]. Gene predictions were then performed using the MetaGeneMark [62]. After the predicted genes were clustered with Cd-hit, a non-redundant gene category was obtained [63]. The information of gene function and species were gained by the gene category mapping to the public databases, including NR, Swiss-Prot, KEGG [64], and gene ontology (GO) [65]. The reads were then aligned against the gene category, and the gene and species abundance of each sample was calculated. Finally, species diversity analysis (Shannon index and comparative analysis), principle component analysis (PCA), GO functional classification analysis, and pathway enrichment analysis performed based on the gene and species abundance data.

For metagenomics *de novo* assembly of each sample, the different k-mer sizes were used to perform multiple *de novo* assembly at the same time and reads then compared with the assembly results to evaluate the assembled effect. Finally, comprehensively to consider the median length of fragments assembled (N50) and mapping rate, the best assembly result of each sample was selected for subsequent analysis.

The analysis of gene expression was performed by using the Reads per Kilobase per Million reads method (RPKM) [66]. A false discovery rate (FDR) ≤ 0.001 [67] and an absolute value of the log (fold-change ratio) ≥ 1 were set as the threshold to identify the significantly differentially expressed genes (DEGs) between the rhizosphere soil of chlorotic and normal plants.

For annotation, all the DEGs were searched against the National Centre for Biotechnology Information (NCBI) for non-redundant protein (NR), Swiss-Prot, EuKaryotic Orthologous Groups of proteins (KOG) (Altschul et al. [64]), and GO databases with a BLAST alignment. The pathway enrichment analysis of DEGs was completed by using Kyoto Encyclopedia of Genes and Genomes [68] All the standard bioinformatics analyses were carried out at the BGI facility.

### Field investigation and pathogen isolation

The absorption roots in the upper, middle, and lower layer of soil were sampled for the pathogen isolations of fungal and bacterial diseases according to reported protocols [37, 69].

### Statistical analysis

Primary data from the soil analyses and relative abundance of bacteria species were subjected to one-way analysis of variance (ANOVA). When differences were found at *P* ≤ 0.05 or *P* ≤ 0.01, least significant difference (LSD) values were calculated to compare the means using Duncan’s multiple range test in SPSS Version 19.0 software for Windows (SPSS Inc., Chicago, IL, USA).

## Supplementary Information

**Additional file 1: Supplementary Figure S1.** Symptoms on ‘Whangkeumbea’ pear leaves of Fe-deficiency chlorotic and normal plants. **Supplementary Figure S2.** Soil layer and root growth investigations at ‘Whangkeumbea’ pear orchard with Fe-deficiency chlorotic and normal plants. **Supplementary Table S1.** Mineral elements in ‘Whangkeumbea’ pear leaves with different degrees of chlorosis. **Supplementary Table S2.** Correlation analysis of mineral elements in ‘Whangkeumbea’ pear leaves with different degree of chlorosis. **Supplementary Table S3.** Number of genes and microbiome annotated in the rhizosphere soil between Fe-deficiency chlorotic and normal plants. **Supplementary Table S4.** DEGs with pathway annotation of Fe-deficiency chlorotic plants. **Supplementary Table S5.** Phyla having a relative abundance greater than 0.5% in any one sample from chlorotic and normal plants. **Supplementary Table S6**. Species with a higher relative abundance and the increment of CH vs. CK

## Data Availability

Sequence data of this project have been deposited in the Sequence Read Archive (SRA) of the National Center for Biotechnology Information (NCBI) under the accession number PRJNA741286 (https://www.ncbi.nlm.nih.gov/bioproject/PRJNA741286).

## Abbreviations

FDC: Fe-deficiency chlorosis
AFe: Available Fe
TFe: Total ferric iron
TEM: Transmission electron microscope
dRi: Degraded ribosome
dST: Destroyed stack of thylakloid
sCh: Smaller chloroplast
lVa: Larger vacuole
Mi: Mitochondria
Ri: Ribosome
ST: Stack of thylakloid
Ch: Chloroplast
Va: Vacuole
CH: Fe-deficiency chlorosis
CK: Control normal plants
OM: Organic matter
CEC: Cation exchange capacity
TFe: Total ferrum
AFe: Available ferrous iron
NN: Nitrate nitrogen
AhN: Alkali hydrolysable nitrogen
AN: Amonium nitrogen
TN: Total nitrogen
AP: Available phosphorus
AK: Available potassium
ECa: Exchangeable calcium
EMg: Exchangeable magnesium
TS: total sulfur
GO: Gene ontology
DEGs: Differentially expressed genes
KEGG: Kyoto Encyclopedia of Genes and Genomes
PCA: Principle component analysis
RPKM: Reads per Kilobase per Million
FDR: False discovery rate
