# Supplementary material for: Metagenomic analysis of rhizosphere microbiome provides insights into occurrence of iron deficiency chlorosis in field of Asian pears
Source: BMC Microbiol. 2022 Jan 8;22:18. doi: 10.1186/s12866-021-02432-7 (PMC8742312; doi:10.1186/s12866-021-02432-7)
Supplement: Supplementary file 1 — Additional file 1: Supplementary Figure S1. Symptoms on ‘Whangkeumbea’ pear leaves of Fe-deficiency chlorotic and normal plants. Supplementary Figure S2. Soil layer and root growth investigations at ‘Whangkeumbea’ pear orchard with Fe-deficiency chlorotic and normal plants. Supplementary Table S1. Mineral elements in ‘Whangkeumbea’ pear leaves with different degrees of chlorosis. Supplementary Table S2. Correlation analysis of mineral elements in ‘Whangkeumbea’ pear leaves with different degree of chlorosis. Supplementary Table S3. Number of genes and microbiome annotated in the rhizosphere soil between Fe-deficiency chlorotic and normal plants. Supplementary Table S4. DEGs with pathway annotation of Fe-deficiency chlorotic plants. Supplementary Table S5. Phyla having a relative abundance greater than 0.5% in any one sample from chlorotic and normal plants. Supplementary Table S6. Species with a higher relative abundance and the increment of CH vs. CK [file 12866_2021_2432_MOESM1_ESM.docx]

# Supplementary Figures & Tables

# Title

**Metagenomic analysis of rhizosphere microbiome provides insights into occurrence of iron deficiency chlorosis in field of Asian pears**

# Authors

Bing Jia^1¶^, Xiao Chang^1¶^, Yuanyuan Fu^1^, Wei Heng^1^, Zhenfeng Ye^1^, Pu Liu^1^, Li Liu^1^, Yosef Al Shoffe^2^, Christopher Brian Watkins^2^ & Liwu Zhu^1, 2*^

^1^ School of Horticulture, Anhui Agricultural University, Hefei 230036, Anhui, P.R. China; ^2^ College of Agriculture and Life Sciences, Cornell University, Ithaca, NY 14853, USA

^¶^These authors contributed equally to this work.

*Corresponding author: E-mail: zhuliwu@ahau.edu.cn; Tel. +86 551 65786607


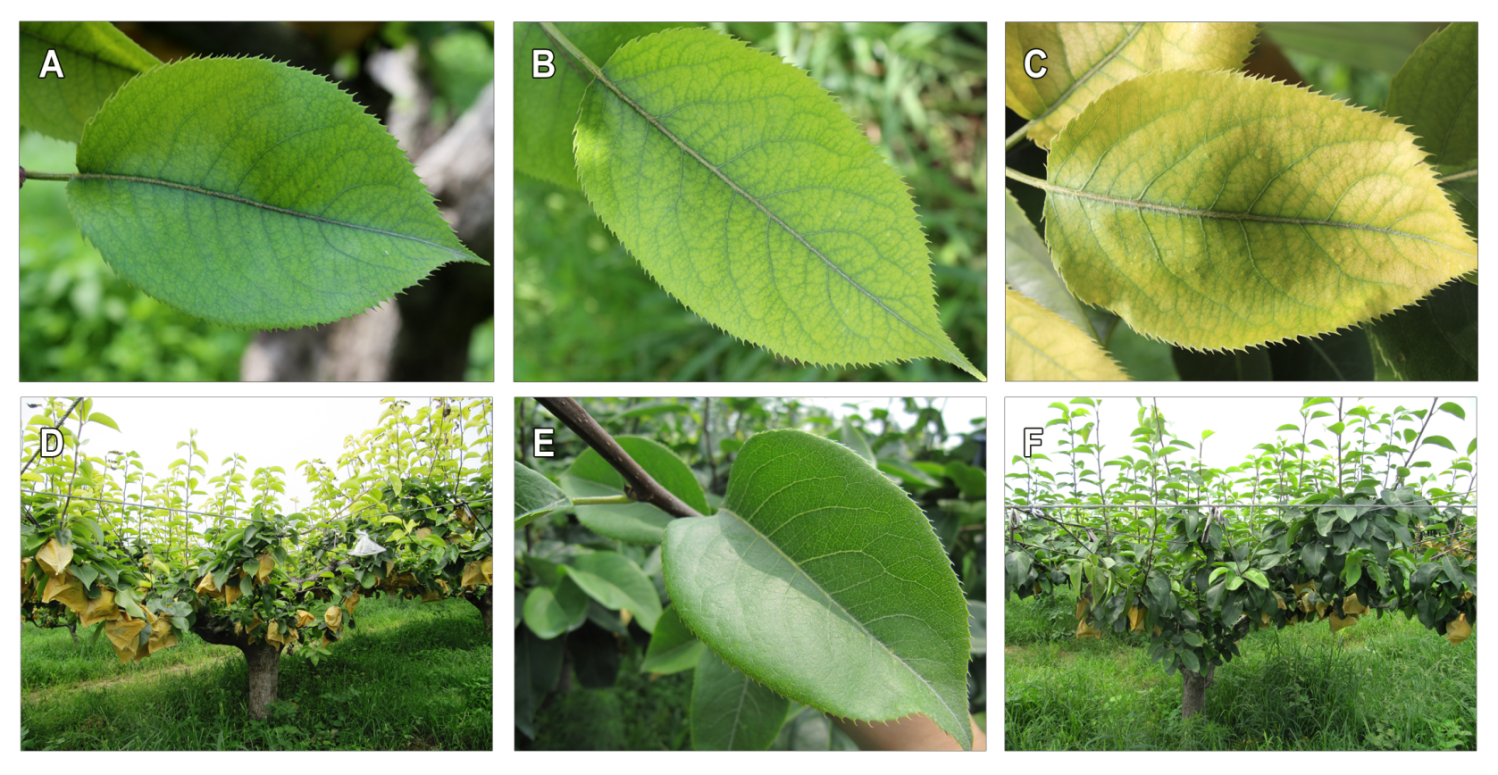


## Supplementary Fig. S1 Symptoms on ‘Whangkeumbea’ pear leaves of Fe-deficiency chlorotic and normal plants

A. leaf with mild Fe-deficiency, lighter-green to lime-green color of interveinal tissue; B. leaf with moderate Fe-deficiency, yellow color of interveinal tissue; C. leaf with severe Fe-deficiency, leaf veins turn yellow; D. Fe-deficiency chlorotic plants; E. normal leaf; F. normal plants.


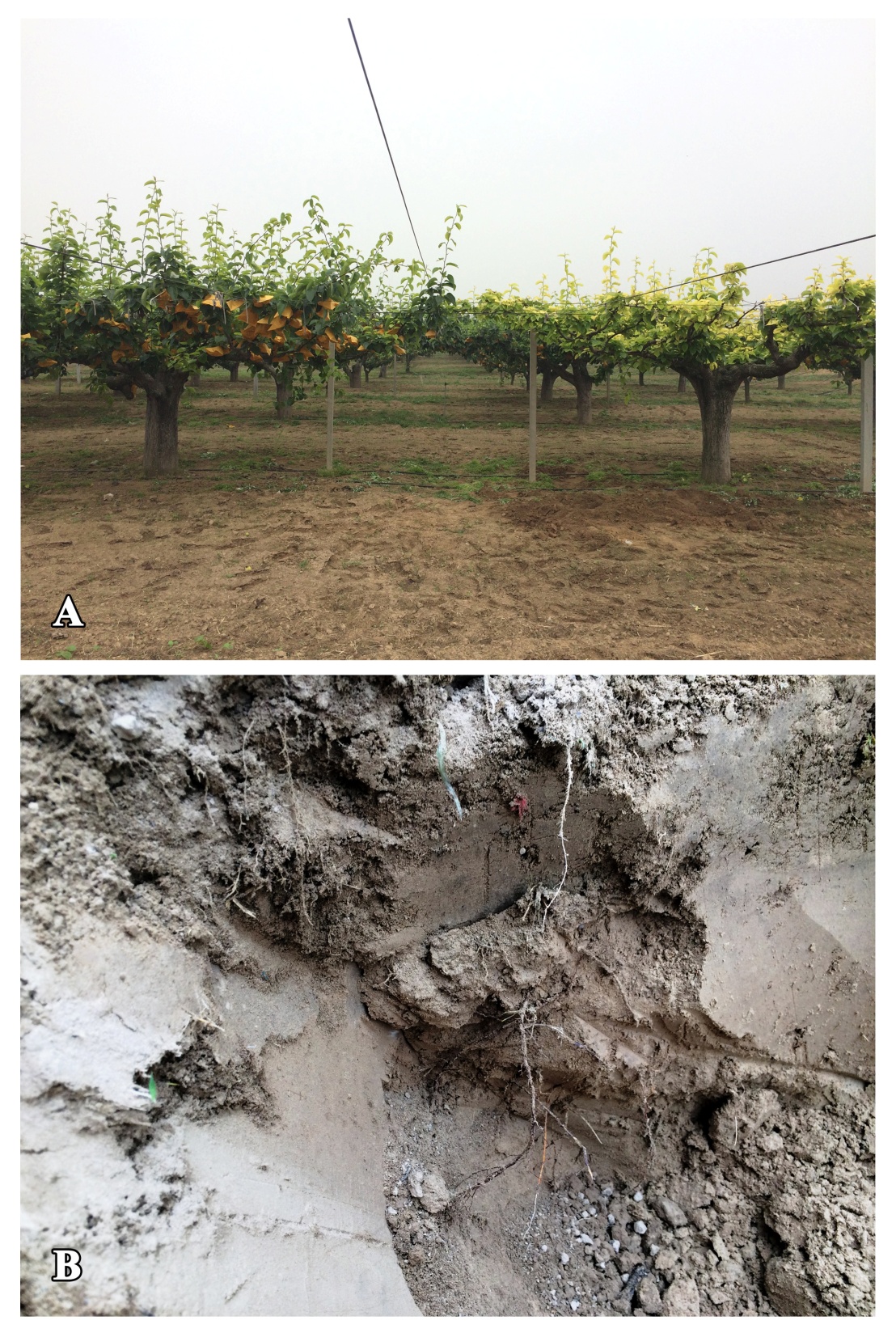


## Supplementary Fig. S2 Soil layer and root growth investigations at ‘Whangkeumbea’ pear orchard with Fe-deficiency chlorotic and normal plants

A. Fe-deficiency chlorotic plants on the right and normal plants on the left; B. A water-impermeable table (10 cm) formed by silt stratification, roots grow normally in the upper layer of soil (20 cm), and some of the absorption roots died from anoxes condition in the middle and lower layers of soil (20 cm).

## Supplementary Table S1 Mineral elements in ‘Whangkeumbea’ pear leaves with different degrees of chlorosis

| **Sample types** | **N**  **(%)** | **P**  **(%)** | **K**  **(%)** | **Ca**  **(%)** | **Mg**  **(%)** | **S**  **(%)** | **Fe**  **(mg/kg)** | **B**  **(mg/kg)** | **Mn**  **(mg/kg)** | **Cu**  **(mg/kg)** | **Zn**  **(mg/kg)** | **Mo**  **(mg/kg)** | **Cl**  **(mg/kg)** |
| --- | --- | --- | --- | --- | --- | --- | --- | --- | --- | --- | --- | --- | --- |
| Severe chlorosis | 1.63 | 0.14 | 2.40 | 2.21 | 0.44 | 0.18 | 91.26 c | 27.04 | 50.61 | 12.95 | 27.4 | 1.22 | 578 |
| Moderate chlorosis | 1.10 | 0.20 | 2.45 | 1.80 | 0.39 | 0.17 | 125.42 bc | 29.47 | 47.54 | 16.91 | 30.97 | 1.45 | 549 |
| Mild chlorosis | 1.64 | 0.34 | 2.68 | 1.30 | 0.47 | 0.18 | 134.40 b | 39.28 | 37.83 | 19.75 | 27.57 | 2.13 | 622 |
| Normal leaves | 2.06 | 0.48 | 3.68 | 1.50 | 0.67 | 0.27 | 328.80 a | 52.40 | 60.91 | 32.97 | 41.39 | 41.39 | 773 |

Note: the deferent lowercase means significant deference at the P value less than 0.05.

## Supplementary Table S2 Correlation analysis of mineral elements in ‘Whangkeumbea’ pear leaves with different degree of chlorosis

| **Correlation** | **N** | **P** | **K** | **Ca** | **Mg** | **S** | **Fe** | **B** | **Mn** | **Cu** | **Zn** | **Mo** | **Cl** |
| --- | --- | --- | --- | --- | --- | --- | --- | --- | --- | --- | --- | --- | --- |
| **N** | 1 | 0.725 | 0.799 | -0.306 | 0.900 | 0.828 | 0.722 | 0.783 | 0.517 | 0.710 | 0.583 | 0.771 | 0.888 |
| **P** | 0.725 | 1 | 0.928 | -0.796 | 0.888 | 0.842 | 0.903 | 0.993 | 0.368 | 0.958 | 0.782 | 0.845 | 0.922 |
| **K** | 0.799 | 0.928 | 1 | -0.517 | 0.980 | 0.981 | 0.992 | 0.963 | 0.686 | 0.986 | 0.932 | 0.983 | 0.985 |
| **Ca** | -0.306 | -0.796 | -0.517 | 1 | -0.435 | -0.345 | -0.487 | -0.718 | 0.244 | -0.622 | -0.327 | -0.359 | -0.507 |
| **Mg** | 0.900 | 0.888 | 0.980* | -0.435 | 1 | 0.983 | 0.951 | 0.937 | 0.694 | 0.936 | 0.871 | 0.966 | 0.997 |
| **S** | 0.828 | 0.842 | 0.981* | -0.345 | 0.983* | 1 | 0.974 | 0.899 | 0.799 | 0.939 | 0.938 | 0.995 | 0.973 |
| **Fe** | 0.722 | 0.903 | 0.992** | -0.487 | 0.951* | 0.974* | 1 | 0.938 | 0.726 | 0.987 | 0.969 | 0.988 | 0.955 |
| **B** | 0.783 | 0.993** | 0.963* | -0.718 | 0.937 | 0.899 | 0.938 | 1 | 0.465 | 0.975 | 0.827 | 0.897 | 0.962 |
| **Mn** | 0.517 | 0.368 | 0.686 | 0.244 | 0.694 | 0.799 | 0.726 | 0.465 | 1 | 0.606 | 0.829 | 0.808 | 0.643 |
| **Cu** | 0.710 | 0.958* | 0.986* | -0.622 | 0.936 | 0.939 | 0.987* | 0.975* | 0.606 | 1 | 0.928 | 0.953 | 0.953 |
| **Zn** | 0.583 | 0.782 | 0.932 | -0.327 | 0.871 | 0.938 | 0.969* | 0.827 | 0.829 | 0.928 | 1 | 0.967 | 0.865 |
| **Mo** | 0.771 | 0.845 | 0.983* | -0.359 | 0.966* | 0.995** | 0.988* | 0.897 | 0.808 | 0.953* | 0.967* | 1 | 0.958 |
| **Cl** | 0.888 | 0.922 | 0.985* | -0.507 | 0.997** | 0.973* | 0.955* | 0.962* | 0.643 | 0.953* | 0.865 | 0.958* | 1 |

Note: * Correlation is significant at the 0.05 level (2-tailed), ** Correlation is significant at the 0.01 level (2-tailed).

## Supplementary Table S3 Number of genes and microbiome annotated in the rhizosphere soil between Fe-deficiency chlorotic and normal plants

| Sample Name | Raw  Reads | Clean Reads | Total Gene | Annotated Gene | Annotated Kingdom | Annotated Phylum | Annotated Class | Annotated Order | Annotated Family | Annotated Genus | Annotated Species |
| --- | --- | --- | --- | --- | --- | --- | --- | --- | --- | --- | --- |
|  |  |  |  |  |  |  |  |  |  |  |  |
| CK1 | 52164932 | 47612900 | 80594 | 61004 | 5 | 53 | 93 | 203 | 401 | 1169 | 3021 |
| CK2 | 53881466 | 49194716 | 55664 | 44323 | 5 | 54 | 94 | 205 | 386 | 1048 | 2525 |
| CK3 | 53881226 | 48835220 | 61220 | 48077 | 5 | 53 | 96 | 206 | 392 | 1089 | 2644 |
| Average | 53309208 | 48547612 | 65826 | 51135 | 5 | 53 | 94 | 205 | 393 | 1102 | 2730 |
| CH1 | 53881202 | 49387458 | 77249 | 60238 | 5 | 52 | 94 | 207 | 406 | 1147 | 2987 |
| CH2 | 44167196 | 40289542 | 62062 | 49617 | 5 | 51 | 93 | 201 | 393 | 1087 | 2687 |
| CH3 | 53881062 | 49364110 | 114503 | 83097 | 5 | 53 | 96 | 211 | 414 | 1249 | 3456 |
| Average | 50643153 | 46347037 | 84605 | 64317 | 5 | 52 | 94 | 206 | 404 | 1161 | 3043 |

Note: CK1-3. rhizosphere soil of normal plants; CH1-3. rhizosphere soil of chlorotic plants.

## Supplementary Table S4 DEGs with pathway annotation of Fe-deficiency chlorotic plants

| **No.** | **Pathway** | **Number of DEGs** | **Accounting for percentage of total DEGs** | **P value** | **Pathway ID** |
| --- | --- | --- | --- | --- | --- |
|  | [Metabolic pathways](#RANGE!gene9) | 1090 | 33.94 | 0.0102 | ko01100 |
|  | [Biosynthesis of secondary metabolites](#RANGE!gene17) | 495 | 15.41 | 0.0298 | ko01110 |
|  | [Purine metabolism](#RANGE!gene3) | 159 | 4.95 | 0.0041 | ko00230 |
|  | [Alanine, aspartate and glutamate metabolism](#RANGE!gene7) | 77 | 2.40 | 0.0067 | ko00250 |
|  | [Homologous recombination](#RANGE!gene16) | 47 | 1.46 | 0.0260 | ko03440 |
|  | [Mismatch repair](#RANGE!gene15) | 42 | 1.31 | 0.0258 | ko03430 |
|  | [Toluene degradation](#RANGE!gene20) | 11 | 0.34 | 0.0416 | ko00623 |
|  | [Apoptosis](#RANGE!gene2) | 6 | 0.19 | 0.0035 | ko04210 |
|  | [Ethylbenzene degradation](#RANGE!gene12) | 5 | 0.16 | 0.0233 | ko00642 |
|  | [Biosynthesis of siderophore group nonribosomal peptides](#RANGE!gene22) | 4 | 0.12 | 0.0453 | ko01053 |

## Supplementary Table S5 Phyla having a relative abundance greater than 0.5% in any one sample from chlorotic and normal plants

| **Phyla** | **CK1** | **CK2** | **CK3** | **Mean of CK** | **CH1** | **CH2** | **CH3** | **Mean of CH** | **P value** |
| --- | --- | --- | --- | --- | --- | --- | --- | --- | --- |
| Proteobacteria | 48.408 | 49.271 | 44.868 | 47.515 | 48.426 | 44.211 | 35.427 | 42.688 | 0.330 |
| Unclassfied | 29.992 | 28.519 | 31.261 | 29.924 | 27.199 | 27.803 | 36.803 | 30.601 | 0.859 |
| Thaumarchaeota | 3.513 | 4.707 | 5.010 | 4.410 | 13.630 | 18.593 | 5.120 | 12.448 | 0.128 |
| Gemmatimonadetes | 3.907 | 3.611 | 3.783 | 3.767 | 0.463 | 0.534 | 2.893 | 1.297 | 0.037 |
| Nitrospirae | 0.932 | 2.822 | 3.778 | 2.511 | 1.049 | 0.940 | 0.634 | 0.874 | 0.057 |
| Actinobacteria | 2.745 | 2.240 | 2.217 | 2.401 | 2.058 | 1.626 | 2.474 | 2.053 | 0.312 |
| Firmicutes | 1.858 | 2.033 | 2.188 | 2.026 | 1.560 | 1.470 | 2.250 | 1.760 | 0.402 |
| Acidobacteria | 2.176 | 1.259 | 1.347 | 1.594 | 1.558 | 1.099 | 6.812 | 3.156 | 0.491 |
| Others | 1.580 | 1.450 | 1.550 | 1.526 | 1.239 | 1.312 | 1.560 | 1.370 | 0.229 |
| Cyanobacteria | 1.383 | 1.232 | 1.250 | 1.289 | 1.094 | 0.973 | 1.937 | 1.335 | 0.900 |
| Bacteroidetes | 1.290 | 0.908 | 0.952 | 1.050 | 0.669 | 0.434 | 1.388 | 0.830 | 0.549 |
| Planctomycetes | 0.563 | 0.437 | 0.463 | 0.487 | 0.376 | 0.320 | 1.118 | 0.605 | 0.708 |
| Crenarchaeota | 0.202 | 0.750 | 0.469 | 0.474 | 0.067 | 0.121 | 0.042 | 0.077 | 0.024 |
| Chloroflexi | 0.488 | 0.363 | 0.372 | 0.408 | 0.272 | 0.265 | 0.847 | 0.461 | 0.819 |
| Verrucomicrobia | 0.375 | 0.371 | 0.383 | 0.376 | 0.278 | 0.262 | 0.561 | 0.367 | 0.938 |
| Ignavibacteriae | 0.589 | 0.029 | 0.109 | 0.242 | 0.063 | 0.037 | 0.133 | 0.078 | 0.305 |

Note: Note: CH1-3. rhizosphere soil of chlorotic plants; CK1-3. rhizosphere soil of normal plants. There was a significant difference of the average relative abundance between CH and CK, when the P value was less than 0.05.

## Supplementary Table S6 Species with a higher relative abundance and the increment of CH vs. CK

| Species | CK1 | CK2 | CK3 | Mean | CH1 | CH2 | CH3 | Mean | Increased percentage  of CH vs. CK | P value |
| --- | --- | --- | --- | --- | --- | --- | --- | --- | --- | --- |
| Unclassfied | 69.75 | 67.92 | 67.89 | 68.52 | 72.63 | 72.89 | 64.97 | 70.16 | 2.3 | 0.7111 |
| Others | 21.31 | 19.97 | 19.18 | 20.16 | 18.53 | 17.89 | 21.48 | 19.30 | -4.24 | 0.7075 |
| Uncultured bacterium | 1.75 | 1.76 | 1.82 | 1.78 | 1.41 | 1.31 | 4.70 | 2.47 | 28.0 | 0.6944 |
| *Candidatus Nitrosoarchaeum koreensis* | 0.86 | 0.06 | 0.53 | 0.48 | 3.03 | 3.14 | 1.03 | 2.40 | 80.0 | 0.0328 |
| *Gemmatirosa kalamazoonesis* | 2.34 | 2.05 | 2.09 | 2.16 | 0.29 | 0.32 | 1.93 | 0.85 | -60.83 | 0.0424 |
| *Candidatus Nitrosoarchaeum limnia* | 0.23 | 0.10 | 0.22 | 0.18 | 0.97 | 1.18 | 0.37 | 0.84 | 78.0 | 0.0288 |
| *Nitrospira defluvii* | 0.80 | 2.68 | 3.63 | 2.37 | 0.91 | 0.80 | 0.50 | 0.74 | -68.96 | 0.2269 |
| *Candidatus Solibacter usitatus* | 0.51 | 0.28 | 0.32 | 0.37 | 0.26 | 0.16 | 1.59 | 0.67 | 44.5 | 0.6878 |
| *Candidatus Nitrosopumilus salaria* | 0.12 | 0.03 | 0.11 | 0.09 | 0.63 | 0.89 | 0.22 | 0.58 | 84.5 | 0.0380 |
| *Chloracidobacterium thermophilum* | 0.25 | 0.12 | 0.20 | 0.19 | 0.22 | 0.11 | 1.20 | 0.51 | 62.2 | 0.4887 |
| *Candidatus Koribacter versatilis* | 0.23 | 0.16 | 0.15 | 0.18 | 0.31 | 0.28 | 0.69 | 0.42 | 57.9 | 0.1117 |
| *Nitrososphaera viennensis* | 0.86 | 3.73 | 2.74 | 2.44 | 0.29 | 0.50 | 0.16 | 0.32 | -87.07 | 0.1147 |
| *Sulfuricella denitrificans* | 0.27 | 0.57 | 0.50 | 0.45 | 0.34 | 0.36 | 0.18 | 0.29 | -34.66 | 0.3643 |
| Uncultured *Acidobacteria bacterium* | 0.16 | 0.09 | 0.11 | 0.12 | 0.12 | 0.07 | 0.58 | 0.26 | 53.9 | 0.5443 |
| *Gemmatimonas aurantiaca* | 0.55 | 0.49 | 0.51 | 0.51 | 0.07 | 0.10 | 0.42 | 0.20 | -61.43 | 0.0227 |

Note: Note: CH1-3. rhizosphere soil of chlorotic plants; CK1-3. rhizosphere soil of normal plants. There was a significant difference of the average relative abundance between CH and CK, when the P value was less than 0.05.
